# The Other Caregivers: Informal Non-Spousal Male Caregivers for Persons With Dementia

**DOI:** 10.1093/geroni/igab046.2929

**Published:** 2021-12-17

**Authors:** Gretchen Tucker

**Affiliations:** University of Maryland, Baltimore, Columbia, Maryland, United States

## Abstract

Informal caregivers for persons with Alzheimer's disease and related dementias (ADRD) have become an integral part of the long-term health care system. They are relied on to provided day-to-day care that is challenging, complex, and often spans several years. Most of the research on informal caregivers for persons with ADRD have focused on spousal caregiving, mother-daughter dyads, and daughters. There is sparse literature on informal non-spousal male caregivers for persons with ADRD. The objective of this research was to obtain an understanding of the experiences of informal non-spousal male caregivers for persons with ADRD. This descriptive qualitative pilot study consisted of in-depth one-on-one interviews with three informal non-spousal male caregivers for persons with ADRD. Four themes emerged through data analysis: 1) the male perspective and experience of caregiving, 2) relationship dynamics, 3) caregiving challenges, and 4) finding meaning within caregiving. Conclusion: Similar to other caregivers, informal non-spousal male caregivers assisted with transportation, managing medical appointments, as well as bathing and personal care. Differences with other caregivers, specifically female caregivers, emerged in terms of descriptions of traditional versus non-traditional gender roles. The implications of this study are that public policies, support services and medical professionals need to understand and be able to address the different experiences and needs of informal non-spousal male caregivers.

